# Puerarin ameliorates nonalcoholic fatty liver in rats by regulating hepatic lipid accumulation, oxidative stress, and inflammation

**DOI:** 10.3389/fimmu.2022.956688

**Published:** 2022-07-25

**Authors:** Jingxuan Zhou, Nanhai Zhang, Adil Aldhahrani, Mohamed Mohamed Soliman, Liebing Zhang, Feng Zhou

**Affiliations:** ^1^ Beijing Key Laboratory of Functional Food from Plant Resources, College of Food Science and Nutritional Engineering, China Agricultural University, Beijing, China; ^2^ Clinical Laboratory Sciences Department, Turabah University College, Taif University, Taif, Saudi Arabia

**Keywords:** nonalcoholic fatty liver disease, puerarin, lipid accumulation, oxidative stress, inflammation

## Abstract

Nonalcoholic fatty liver disease (NAFLD) has become one of the public health problems globally. The occurrence of NAFLD is usually accompanied by a series of chronic metabolic diseases, with a prevalence rate is 25.24% among adults worldwide. Therefore, NAFLD seriously affects the quality of life in patients and causes a large economic burden. It has been reported that puerarin has the function of lowering the serum lipids, but due to the complexity of NAFLD, the specific mechanism of action has not been clarified. The aim of this study was to evaluate the preventive or ameliorating effects of two doses of puerarin (0.11% and 0.22% in diet) on high-fat and high-fructose diet (HFFD)-induced NAFLD in rats. The rats were fed with HFFD-mixed puerarin for 20 weeks. The results showed that puerarin ameliorated the levels of lipids in the serum and liver. Further exploration of the mechanism found that puerarin ameliorated hepatic lipid accumulation in NAFLD rats by reducing the expression of *Srebf1*, *Chrebp*, *Acaca*, *Scd1, Fasn*, *Acacb*, *Cd36*, *Fatp5*, *Degs1*, *Plin2*, and *Apob100* and upregulating the expression of *Mttp*, *Cpt1a*, and *Pnpla2*. At the same time, after administration of puerarin, the levels of antioxidant markers (superoxide dismutase, glutathione peroxidase, and catalase) were significantly increased in the serum and liver, and the contents of serum and hepatic inflammatory factors (interleukin-18, interleukins-1β, and tumor necrosis factor α) were clearly decreased. In addition, puerarin could ameliorate the liver function. Overall, puerarin ameliorated HFFD-induced NAFLD by modulating liver lipid accumulation, liver function, oxidative stress, and inflammation.

## Introduction

Nonalcoholic fatty liver disease (NAFLD) has become the leading cause of chronic liver disease globally, with an estimated global adult prevalence of 25.24% (1, 2). About 20%–30% patients of the NAFLD will develop nonalcoholic hepatitis, and 20% cases of nonalcoholic hepatitis will develop liver cirrhosis, which greatly increases the risk of hepatocellular carcinoma (3). NAFLD was not only accompanied by steatosis, based on the clinical characteristics of majority patients, but is also commonly associated with obesity, dyslipidemia, and type 2 diabetes, and considered a hepatic manifestation of metabolic syndrome (4, 5). Excessive triglycerides (TGs) accumulation in the liver is an important cause for NAFLD (6, 7). The accumulation of TG in hepatocytes is related to four mechanisms: fatty acid uptake, *de novo* lipogenesis, fatty acid oxidation, and very low-density lipoprotein (VLDL) assembly and secretion (8).

After high-fat and high-fructose diet (HFFD), 30% of the fatty acids are transported to the liver tissue (9). The liver can also generate acetyl-CoA through glycolysis by itself and then gradually generate fatty acids by a series of fatty acid synthases. The results of the study showed that 26% of TG in the liver of NAFLD patients were synthesized from *de novo* fatty acids (10). The hepatic fatty acid oxidation mechanism can efficiently consume free fatty acids (FFA) that are synthesized by *de novo* in the liver or obtained in the blood (11). Therefore, FFA β-oxidation is an important way to regulate the homeostasis of hepatic TG. The secretion of VLDL is the only effective way to export TG from the liver (9). The imbalance of the above four mechanisms is the main reason for the excessive accumulation of TG in the liver. Steatosis caused by fat accumulation activates the c-Jun N-terminal kinase signaling pathway, promoting the production of excess reactive oxygen species (ROS) in the mitochondria, which ultimately cause oxidative stress (12). On the other hand, excessive fatty acid oxidation is also a potential pathway for ROS generation (13). During the development of NAFLD, oxidative stress activates some inflammatory cells, which contribute to the development of nonalcoholic steatohepatitis (14). They induce the occurrence of non-alcoholic hepatitis through hepatocyte apoptosis, insulin resistance, and other pathways (15). Therefore, NAFLD is closely related to oxidative stress and inflammatory response. At present, drug treatment of chronic liver disease has become the most common method. Fenofibrate can reduce lipid accumulation in the liver, and statins can reduce the contents of the serum lipids (16, 17). Although they have a significant effect, their long-term administration can trigger hepatotoxicity and nephrotoxicity (18, 19). Since there is no specific drug for NAFLD, only a combination of drugs can be used to regulate the liver injury caused by NAFLD. At the same time, the side effects of drugs are obvious. Therefore, finding a low-toxic or non-toxic natural active ingredient to improve NAFLD has become a hot topic.

Puerarin, a natural active ingredient extracted from *Pueraria lobata*, has antioxidant, anti-inflammation, and insulin resistance-reducing effects (20, 21). According to reports, puerarin has a certain therapeutic effect on hypertension, diabetes, arteriosclerosis, and other diseases in diabetic rats (22, 23). On this basis, more scholars have studied the mechanism of action of puerarin in improving liver steatosis. Studies have found that puerarin could regulate leptin signaling through the Janus kinase 2/signal transducer and activator of transcription 3 signaling pathway, thereby improving liver steatosis (24). Wenting Xu et al. (25) constructed a diabetic rat model by intraperitoneal injection of streptozotocin and found that the puerarin treatment group could significantly reduce the serum TG and TC contents, and the protein expression of inflammatory factors in the puerarin treatment group was significantly lower, such as tumor necrosis factor α (TNF-α). According to recent studies, puerarin could ameliorate glucose content and lipid metabolic disorders in the liver by regulating the adenosine 5′-monophosphate-activated protein kinase pathway and downregulating the gene expression levels of acetoacetyl coenzyme A (Acetyl-CoA) carboxylase (ACC1), fatty acid synthase (FASN), stearoyl-coenzyme A desaturase 1 (SCD1), and sterol regulatory element binding transcription factor-1c (SREBP-1c) (26).

Due to the complexity of the pathogenesis of NAFLD, the research on the mechanism of puerarin in improving NAFLD caused by HFFD is not comprehensive enough, and the regulatory mechanism is not yet clear. In this study, we established a NAFLD rats model induced by HFFD and comprehensively evaluated its improvement effect through serum and liver lipid indexes, antioxidant markers, and inflammatory factors. In addition, the mechanism of action of puerarin in improving NAFLD was explored by detecting the gene expression of related pathways of four mechanisms: *de novo* lipogenesis, fatty acid uptake, fatty acid β-oxidation, and VLDL assembly and secretion.

## Materials and methods

### Chemicals and reagents

Puerarin (purity 98%) was purchased from Nanjing Jingzhu Bio-technology Co., Ltd. (Nanjing, China). Fenofibrate was bought from Recipharm Fontaine (France). The commercial kits of total cholesterol (TC), TG, aspartate transferase (AST), alkaline phosphatase (ALP), and alanine transaminase (ALT) were obtained from Biosino Bio-Technology and Science Inc. (Beijing, China). Enzyme-linked immunosorbent assay (ELISA) kits of FFA, VLDL, acetyl-CoA, acetoacetate (AcAc), and succinate dehydrogenase (SDH) were purchased from Beijing Sino-UK Institute of Biological Technology (Beijing, China). The assay kits of superoxide dismutase (SOD), glutathione peroxidase (GSH-Px), catalase (CAT), malondialdehyde (MDA), and enzyme-linked immunosorbent assay (ELISA) kits of TNF-α, interleukin-18 (IL-18) and interleukin-1β (IL-1β) were bought from Beijing Sino-UK Institute of Biological Technology (Beijing, China). TRIpure reagent was obtained from Aidlab Biotechnologies Co., Ltd. (Beijing, China). FastQuant RT Kit and SuperReal PreMix Plus with SYBR Green were purchased from Tiangen Biotech Co. Ltd. (Beijing, China). The primers of quantitative real-time PCR (qRT-PCR) were synthesized by Sangon Biotech Co., Ltd. (Shanghai, China).

### Animals and diet

A total of 40 male Sprague–Dawley rats (180–220 g, 8 weeks old) were furnished by Beijing Vital River Laboratory Animal Technology Co., Ltd. (Beijing, China) [Certificate SCXK (Beijing) 2016-0006]. All the rats were housed in the animal room maintained at 22 ± 2°C and 45 ± 5% for relative humidity with a standard 12-h light/dark cycle. In addition, the rats were placed in individual cages with food and water *ad libitum*. All animal procedures were performed in accordance with the Animal Ethics Committee of the Beijing Key Laboratory of Functional Food from Plant Resources and the guidelines for the care and use of laboratory animals of the National Institutes of Health.

After the 1-week acclimation period, the rats were randomly divided into five groups (n = 10 per group): normal group (NG), fed with regular rodent diet; model group (MG), fed with HFFD; fenofibrate group (FG), fenofibrate (0.15%) was administered in chow *ad libitum* in the HFFD; low dose of puerarin group (PL), fed with a HFFD supplemented with 0.1% puerarin; and high dose of puerarin group (PH), fed with an HFFD supplemented with 0.2% puerarin. The HFFD and the dose of puerarin were added by referring to previous research studies (27, 28). The dose of fenofibrate was added to the feed according to the study by Laura Castiglioni et al. (29). The compositions of the basal diet and HFFD are shown in **Table 1**. The changes in food intake and body weight were measured once every 2 days and 1 week, respectively. The animal experiment lasted for 20 weeks.

**Table 1:** Composition of diets.

Reagents	Basal diet	HFFD	HFFD+0.11% puerarin	HFFD+0.22% puerarin	HFFD+fenofibrate
Corn powder (g/100 g)	25.0	–	–	–	–
Wheat middling (g/100 g)	20.0	–	–	–	–
Wheat flour (g/100 g)	21.9	42.0	41.9	41.8	40.5
Soybean meal (g/100 g)	20.0	8.0	8.0	8.0	8.0
Chicken powder (g/100 g)	–	8.0	8.0	8.0	8.0
Fructose (g/100 g)	–	18.0	18.0	18.0	18.0
Lard (g/100 g)	–	20.0	20.0	20.0	20.0
Sodium cholate (g/100 g)	–	0.2	0.2	0.2	0.2
Calcium bicarbonate (g/100 g)	1.0	1.0	1.0	1.0	1.0
Mountain flour (g/100 g)	1.6	0.8	0.8	0.8	0.8
Miscellaneous meal (g/100 g)	4.5	–	–	–	–
Plant oil (g/100 g)	2.0	–	–	–	–
Fish powder (g/100 g)	2.0	–	–	–	–
Vitamin mix (g/100 g)	2.0	2.0	2.0	2.0	2.0
Puerarin (g/100 g)	–	–	0.1	0.2	–
Fenofibrate (g/100 g)	–	–	–	–	1.5
Energy (kJ/g)	13.9	18.9	18.9	18.9	18.9

### Measurement of serum and hepatic biochemical indicators

On the 8th, 12th, 16th, and 20th weeks of the treatment, the rats were fasted overnight with water *ad libitum* and anesthetized on the second day. The blood samples were obtained from orbital venous plexus and placed for 12 h at 4°C and then centrifuged at 4,000 *g* for 10 min at 4°C to obtain the serum. Afterward, the serum was stored at −80°C. Blood samples taken in the first three sessions were used to monitor changes in serum lipids. The blood samples at the 20th week were used for the comprehensive detection of serum markers. At the end of the animal experiment, the liver simples were divided into two parts, one part soaked in 10% formalin solution for histopathology and another part stored at −80 °C for biochemical determination. Liver lipids were extracted from liver homogenates referring to the protocols of Zhao et al. (30).

The contents of the serum lipids (TG, FFA, and VLDL) and the hepatic lipids (TC, TG, FFA, and VLDL) were determined using the corresponding detection kits. The levels of liver function indicators (AST, ALP, and ALT) were measured following the instructions of the corresponding assay kits. The variation of antioxidant markers (SOD, CAT, and GSH-Px) and content of MDA in the serum and liver were determined by the corresponding assay kits. The levels of inflammatory cytokines (IL-1β, IL-18, and TNF-α) in the serum and liver, and the levels of lipid metabolites (SDH, AcAc, and acetyl-CoA) in the liver were determined using the corresponding detection kits. All the indicators were detected by a Mindray BS-420 automatic biochemistry analyzer (Shenzhen Mindray Bio-Medical Electronics Co., Ltd., Shenzhen, China).

### Organ and fat coefficient

At the end of the animal experiment, the rats were fasted for 12 h, and the last body weight was measured. Then, all rats were sacrificed under deep anaesthesia. After that, the liver, spleen, kidney, epididymal, perirenal, and abdominal fats were removed and weighted immediately to calculate the organ and fat indexes [organ (fat) index (%) = organ (fat) weight (g)/final body weight (g) × 100%].

### Histological analysis

The liver tissues were soaked in 10% formalin solution for 24 h and cut into slices before embedded in the paraffin. Then, liver sections were stained with hematoxylin and eosin (H&E). After all, the histological changes were observed by a light microscope (BA-9000, Osaka, Japan).

### Quantitative real-time PCR analysis for mRNA expression

The total RNA of the liver was extracted by using TRIpure reagent. The concentration and purity of RNA was assessed by an ultraviolet–visible spectrophotometer (DS-11, Denovix, USA). Then, cDNA was reverse transcribed from RNA by a FastQuant RT Kit. The mRNA expression levels were determined using the SYBR Green system (Bio-Rad Laboratories, Inc., USA), and the *Gapdh* was regarded as an internal reference to normalize mRNA expression. Primer sequences are shown in **Table 2**. The relative mRNA expression levels were calculated *via* the 2^−(ΔΔCt)^ method (31).

**Table 2:** Primer sequence of genes used for qRT-PCR.

Gene	Forward Primer (5′–3′)	Reverse Primer (5′–3′)
*Acaca*	GACAAACGAGTCTGGCTACTAC	CTTGTCTCCATACGCCTGAAA
*Fasn*	ACCTGCTGCTAAAGCCTAAC	GCAATACCCGTTCCCTGAAT
*Srebf1*	CGCTACCGTTCCTCTATCAATG	GCGCAAGACAGCAGATTTATTC
*Chrebp*	GCTGTTGTCTTGGAGGGTAA	GAGCCGCTTCTTGTAGTAGATT
*Scd1*	CGTTCCAGAACGATGTGTATGA	CAACCCACGTGAGAGAAGAAA
*Cd36*	ACGACTGCAGGTCAACATAC	CGATGGTCCCAGTCTCATTTAG
*Fatp5*	TGCCAAGCTTCGTGCTAATA	TGATAGGATGGCTGGCTTTG
*Cpt1a*	GACAAACGAGTCTGGCTACTAC	CTTGTCTCCATACGCCTGAAA
*Acacb*	CTGCTGAGACACAGTAAGGAAC	CCTGGTGAACTCCTCAAATCTC
*Ppara*	TGAAGCAGATGACCTGGAAAG	TCTCCGAGGGACTGAGAAAT
*Pnpla2*	TCAACCTGCGCAATCTCTAC	TAGCCCTGTTTGCACATCTC
*Des1*	GATAACCTCCCACACTACAACTC	CATCCGTGAGTAGGGACTTATTG
*Mttp*	GAAGCAAGTGGCAGGAGTAA	AAGGACAGCCTTTACAGACAC
*Apob100*	CCATATCCCAGACAACCTCTTC	CACCCAAAGGCAAAGGAATG
*Plin2*	CTGAGCACATCGAGTCACATAC	GACAAGTTGCAGGTTCCCAAT
*Gapdh*	GATTCCACCCATGGCAAATTC	CTGGAAGATGGTGATGGGATT

### Statistical analysis

All data were presented as mean ± standard deviation (SD). Data normality and homogeneity of variance were analyzed using Shapiro–Wilk test and Levene test, respectively. If the data where normally and homogeneously distributed, then data were analyzed using independent-samples t-test using SPSS 26.0 (SPSS Inc., Chicago, USA). Mann–Whitney U test was used for data analysis when data did not conform to normal or homogeneous distribution. *p*-values <0.05 and 0.01 were considered to be statistically and highly statistically significant, respectively.

## Result

### Effect of puerarin on food intake, body weight, and organ coefficient

**Table 3** shows the changes in body weight, food intake, and coefficients of organ and rats after the animal experiment. The rats in the MG showed a significant increase in final weight, index of liver, and perirenal, and fat of abdominal and epididymal [*p* < 0.05, *vs* NG]. The kidney index in the MG was greatly declined (*p* < 0.05, *vs*. NG). In particular, the body weight and abdominal fat in the MG were increased by 1.27- and 2.83-fold, respectively (*p* < 0.01, *vs* NG). In all the treatment groups, the contents of final weight, perirenal, and abdominal fat were obviously lowered (*p* < 0.01, *vs*. MG). After dietary intervention with puerarin, the liver coefficient of rats was significantly reduced (*p* < 0.01, *vs*. MG). Interestingly, there was no difference in epididymal fat coefficient with a high dose of puerarin, but a low dose of puerarin could reduce epididymal fat coefficient clearly (*p* < 0.01, *vs*. MG). The food intake of rats in the FG was decreased sharply (*p* < 0.01, *vs*. MG). Fenofibrate administration could also reduce the epididymal fat coefficient (*p* < 0.01, *vs*. MG). Surprisingly, the liver coefficient in the FG was 2.53 times that of the MG and 2.84 times that of the NG (*p* < 0.01, *vs*. MG and NG).

**Table 3:** Effects of puerarin on body weight, food intake, and organ and fat coefficient.

Groups	NG	MG	FG	PH	PL
Initial weight (g)	360.00 ± 16.26	359.71 ± 15.48	361.13 ± 17.82	360.75 ± 17.40	359.38 ± 16.25
Final weight (g)	598.67 ± 24.31	761.45 ± 51.95^**^	570.67 ± 32.56^##^	660.67 ± 23.39^**##^	649.00 ± 33.25^**##^
Food intake (g/day)	25.26 ± 2.90	23.53 ± 1.84	18.29 ± 2.35^##**^	22.43 ± 2.00	22.54 ± 2.32
Spleen index (%)	0.13 ± 0.01	0.10 ± 0.01	0.14 ± 0.01^#^	0.11 ± 0.01	0.12 ± 0.01
Liver index (%)	2.13 ± 0.11	2.38 ± 0.12^*^	6.04 ± 0.68^##**^	2.17.14^#^	2.08 ± 0.05^##^
Kidney index (%)	0.51 ± 0.02	0.46 ± 0.02^*^	0.64 ± 0.07^**##^	0.46 ± 0.02^*^	0.46 ± 0.03^*^
Perirenal index (%)	0.49 ± 0.09	1.09 ± 0.10^**^	0.64 ± 0.14^**##^	0.79 ± 0.12^**##^	0.81 ± 0.05^**##^
Abdominal fat (%)	1.51 ± 0.20	4.28 ± 0.54^**^	1.25 ± 0.22^**##^	2.65 ± 0.37^**##^	2.61 ± 0.33^**##^
Epididymal fat (%)	1.61 ± 0.18	2.67 ± 0.36^**^	1.31 ± 0.34^**##^	2.69 ± 0.19^**^	2.41 ± 0.13^**##^

Data were expressed as mean ± SD (n = 10), *p < 0.05, **p < 0.01, vs. NG; ^#^p < 0.05, ^##^p < 0.01, vs. MG.

### Effect of puerarin on serum lipid

**Figure 1:**
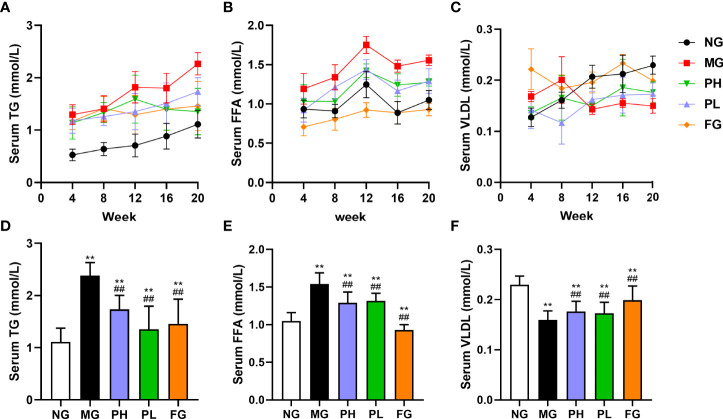
Effect of puerarin on serum lipid. **(A)** Changes in serum TG at 4–20 weeks; **(B)** changes in serum FFA at 4–20 weeks; **(C)** changes in serum VLDL at 4–20 weeks; **(D)** serum TG at 20 weeks; **(E)** serum FFA at 20 weeks; **(F)** serum VLDL at 20 weeks. Data were expressed as mean ± SD (n = 10). **p < 0.01, *vs*. NG; ^##^p < 0.01, *vs*. MG.

As can be seen in **Figures 1A, B**, the levels in the serum TG and FFA were elevated in all rats. The level of FFA in the serum were significantly elevated in all groups at 12 weeks. In **Figure 1C**, the content of serum VLDL in the NG, PH, and PL were gradually increased. The level of serum VLDL in the MG was gradually decreased, and the serum VLDL level of the FG has been in a fluctuating state. The contents of serum FFA and TG in the PH were significantly decreased at week 16 while increasing the serum VLDL content. The PL could decrease serum FFA content at the same time; it did significantly enhance the serum VLDL level at the 12th week. For serum TG, PL increased steadily from the beginning to the end of the experiment, but its content level was far less than that of the MG (*p* < 0.01).

After 20 weeks with HFFD, the levels of serum TG and FFA in the MG were increased by 2.05- and 1.54-fold, respectively (**Figures 1D, E**, *p* < 0.01, *vs*. NG), and the serum VLDL content was decreased by 1.53-fold (**Figure 1F**, *p* < 0.01, *vs*. NG). Compared with the MG, the contents of serum TG and FFA in the PH, PL, and FG were obviously reduced, but the level of serum VLDL was significantly elevated (*p* < 0.01). A low dose of puerarin had the best effect in downregulating serum TG content, which lowered by 30% compared to the MG. Fenofibrate had the best improvement effect on serum FFA and VLDL levels, which decreased by 53.33% and increased by 19.60%, respectively (*vs*. MG).

### Effect of puerarin on hepatic lipid

**Figure 2:**
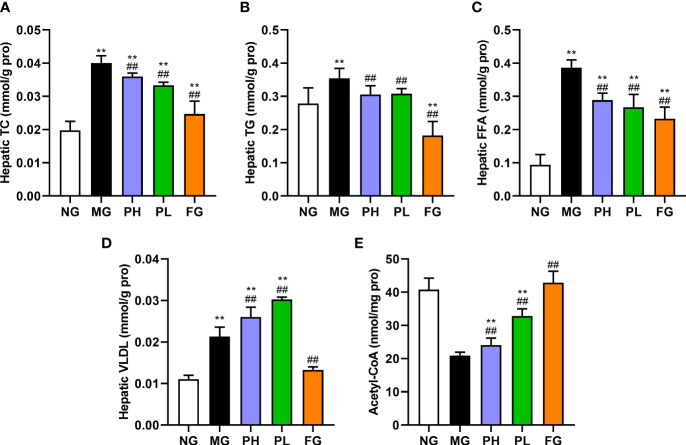
Effect of puerarin on hepatic lipid. **(A)** Hepatic TC; **(B)** hepatic TG; **(C)** hepatic FFA; **(D)** hepatic VLDL; **(E)** hepatic acetyl-CoA. Data were expressed as mean ± SD (n = 10). **p < 0.01, *vs*. NG; ^##^p < 0.01, *vs*. MG.

**Figure 2** shows the hepatic lipid profiles of rats in each group. Compared with the NG, the levels of TC, TG, FFA, and VLDL in the liver showed a clearly upward trend in the MG (**Figures 2A–D**, *p* < 0.01), and the levels of hepatic acetyl-CoA had an obvious decline (**Figure 2E**, *p* < 0.01). Puerarin and fenofibrate could obviously reduce the levels of hepatic TC, TG, and FFA in HFFD rats (*p* < 0.01, *vs*. MG), and the levels of VLDL and acetyl-CoA were significantly enhanced (*p* < 0.01, *vs*. MG). Interestingly, puerarin administration groups instead increased the hepatic VLDL levels (*p* < 0.01, *vs*. MG), which was opposite to the results of the NG and the FG. Overall, fenofibrate had the best effect on regulating hepatic lipids. In addition, the improvement effect of the low dose of puerarin was better than that of the high dose of puerarin. Especially, the content of hepatic TG in the PL was close to that in the NG.

### Effect of puerarin on hepatic function

**Figure 3:**
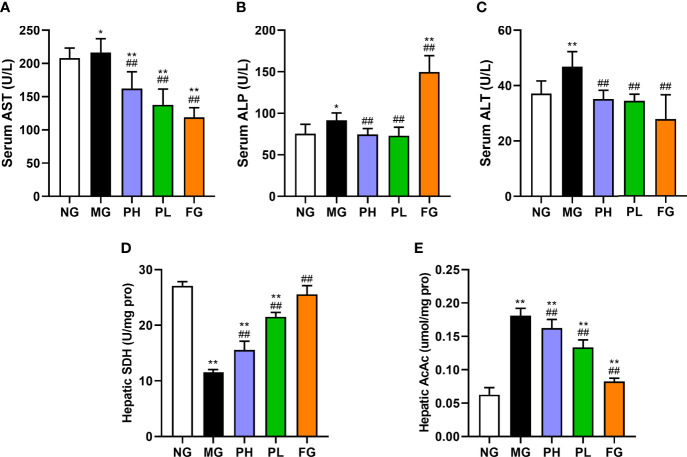
Effect of puerarin on hepatic function. **(A)** Serum AST; **(B)** serum ALP; **(C)** serum ALT; **(D)** hepatic SDH; **(E)** hepatic AcAc. Data were expressed as mean ± SD (n = 10). *p < 0.05, **p < 0.01, *vs*. NG; ^##^p < 0.01, *vs*. MG.

The levels of hepatic AST, ALP, ALT, and AcAc in the MG were significantly higher than those in the NG (**Figure 3**, *p* < 0.01). The levels of AST, ALT, and AcAc in all treatment groups were significantly decreased (*p* < 0.01, *vs*. MG). The ALP levels in the puerarin groups were significantly reduced (*p* < 0.01, *vs*. MG), and the serum ALP level was close to that of the NG. However, the FG showed a significant increase in the level of ALP by 3.17-fold (*p* < 0.01, *vs*. MG). The content of SDH in the PH, PL, and FG was clearly enhance compared to the MG (**Figure 3D**, *p* < 0.01). In puerarin treatment groups, the low dose of puerarin had the best improvement effect on hepatic function.

### Effect of puerarin on oxidative stress

**Figure 4:**
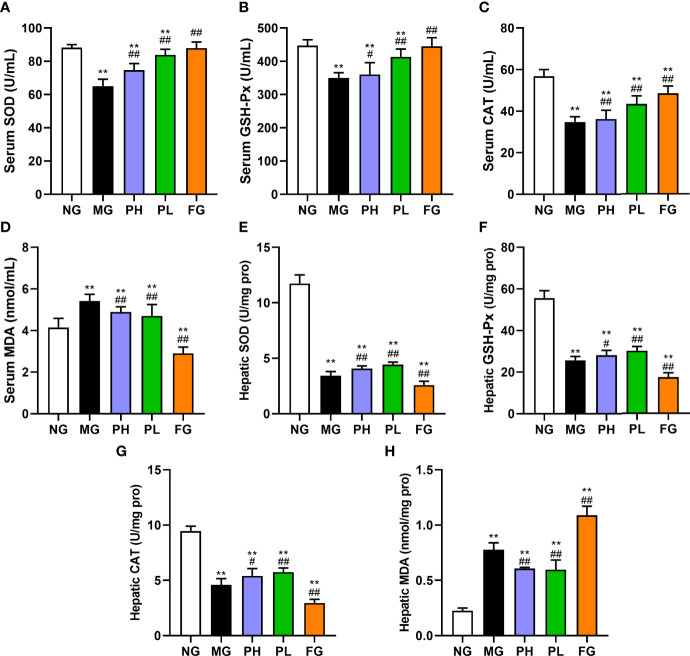
Effect of puerarin on oxidative stress. **(A)** Serum SOD; **(B)** serum GSH-Px; **(C)** serum CAT; **(D)** serum MDA; **(E)** hepatic SOD; **(F)** hepatic GSH-Px; **(G)** hepatic CAT; **(H)** hepatic MDA. Data were expressed as mean ± SD (n = 10). **p < 0.01, *vs*. NG; ^#^p < 0.05, ^##^p < 0.01, *vs*. MG.

The activities of SOD, GSH-Px, and CAT, and the content of MDA in the serum and liver were detected to reflect the influences of puerarin on antioxidant capacity and lipid peroxidation in rats with HFFD (**Figures 4A–H**). The contents of SOD, GSH-Px, and CAT in the serum and liver were markedly decreased in rats treated with pure HFFD, whereas the trend of MDA was reversed (*p* < 0.01, *vs*. NG). The contents of SOD, CAT, and GSH-Px in both the serum and liver in the puerarin groups were visibly higher than those in the MG in both the serum and liver (*p* < 0.01). Among them, a low dose of puerarin has the best effect in all aspects. It is worth noting that the contents of serum and liver antioxidant markers in the FG showed different performances. At the serum level, the SOD, GSH-Px, and CAT in the FG were significantly increased, and the serum MDA content was dropped markedly (*p* < 0.01, *vs*. MG). However, at the liver level, the levels of antioxidant indicators detected were in the opposite trend to those in the serum.

### Effect of puerarin on inflammatory cytokines

**Figure 5:**
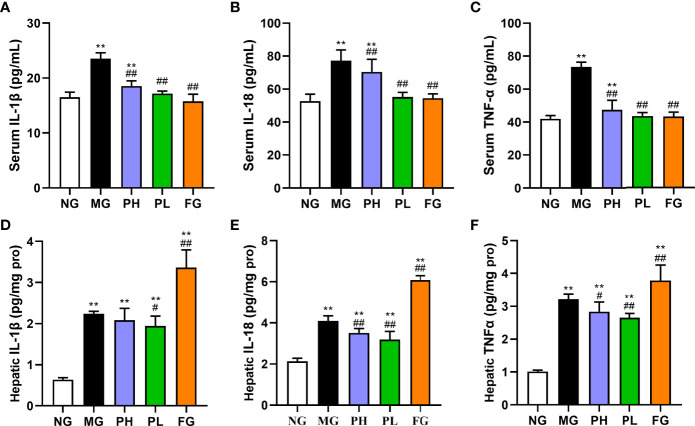
Effect of puerarin on inflammatory cytokines. **(A)** Serum IL-1β; **(B)** serum IL-18; **(C)** serum TNF-α; **(D)** hepatic IL-1β; **(E)** serum IL-18; **(F)** hepatic TNF-α. Data were expressed as mean ± SD (n = 10). *p < 0.05, **p < 0.01, vs. NG; ##p < 0.01, vs. MG.

As shown in **Figure 5**, the levels of IL-1β, IL-6, and TNF-α in both serum and liver were examined to reflect the body inflammation in rats. After the supplementation of HFFD for 20 weeks, the contents of IL-1β, IL-18, and TNF-α in the serum and liver were distinctly increased (**Figures 5A–C**, *p* < 0.01, *vs*. NG). The contents of IL-1β, IL-6, and TNF-α in the serum and liver were all obviously lowered in the PL (*p* < 0.01, *vs*. MG). In addition to the level of liver IL-1β, the high dose of puerarin could significantly reduce the contents of all other inflammatory factors (*p* < 0.05, *vs*. MG). Although fenofibrate administration could significantly decline the contents of serum IL-1β, IL-6, and TNF-α, it did markedly hoist the contents of liver-related indicators (*p* < 0.01, *vs*. MG). Among all treatment groups, the improvement effect of PL was the most obvious, in which serum IL-1β, IL-18, and TNF-α were decreased by 18.87%, 17.93%, and 38.97%, respectively (*vs*. MG).

### Effect of puerarin on histological analysis

**Figure 6:**
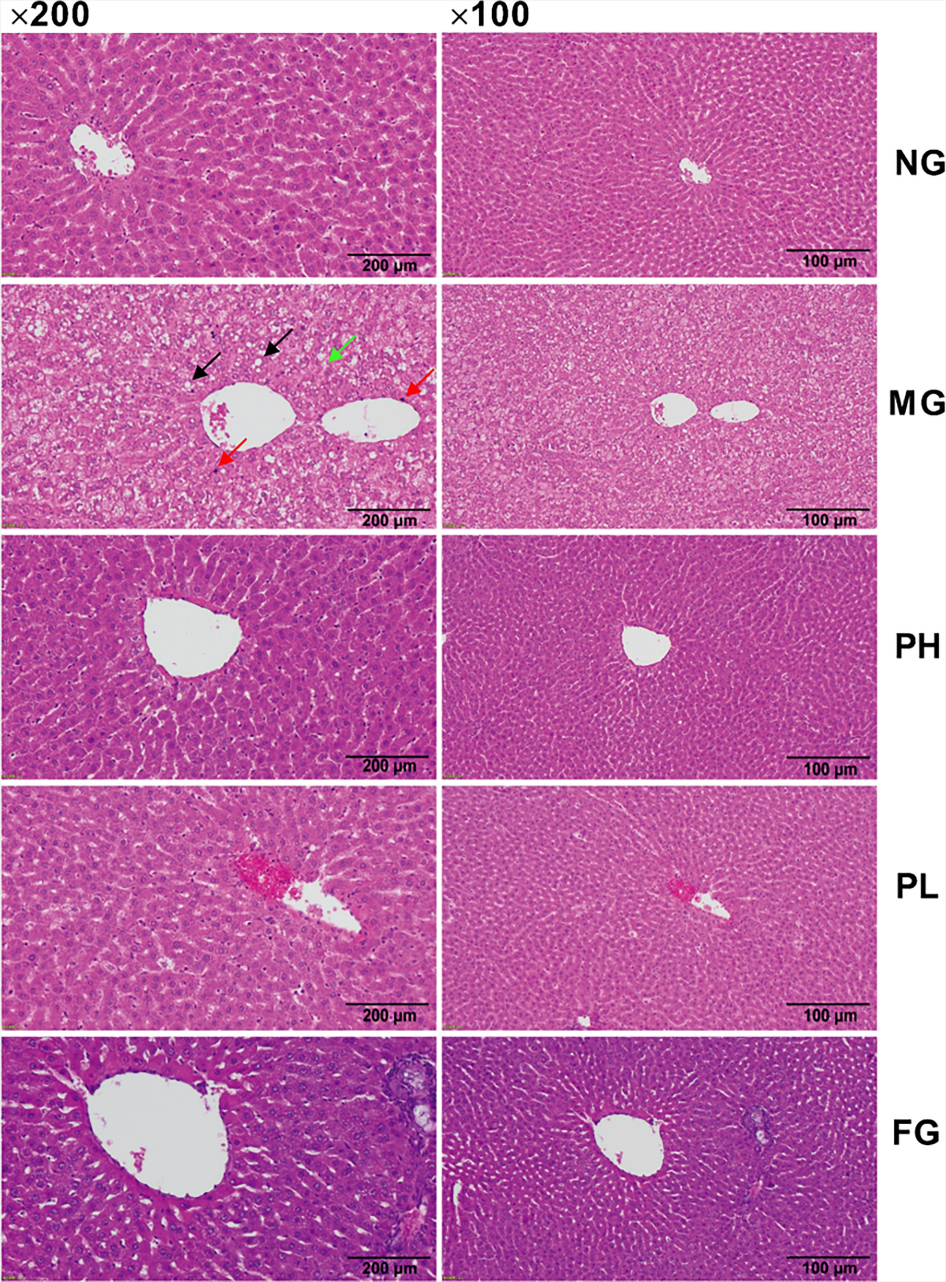
Hematoxylin and eosin (H&E) staining of liver (100× and 200× magnification). The black, red, and green arrows represent fat vacuoles, inflammatory cells, and steatosis.

Histopathological examination of rat liver was performed to determine the role of puerarin in HFFD-induced pathological changes in the liver (**Figure 6**). The hepatocytes in the NG were arranged normally, and there was no steatosis and fat vacuoles. HFFD feeding for 20 weeks caused the number of fatty vacuoles to significantly increase, with steatosis, disordered arrangement of hepatocytes, and more inflammatory cell infiltration. Under the intervention of puerarin, the fatty vacuoles in hepatocytes were clearly reduced, the arrangement of liver cells returned to normal, and there was less inflammatory cell infiltration. Compared with the MG, although the number fatty vacuoles were significantly reduced after fenofibrate administration, the infiltration of inflammatory cells was still more.

### Effect of puerarin on expression of lipid metabolism-related genes in the liver

**Figure 7:**
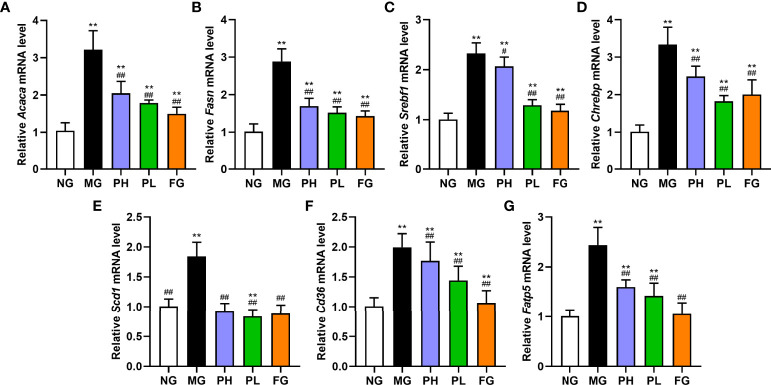
Effect of puerarin on expression of lipid synthesis-related genes in hepatic. **(A)** *Acaca*; **(B)** *Fasn*; **(C)** *Srebf1*; **(D)** *Chrebp*; **(E)** *Scd1*; **(F)** *Cd36*; **(G)** *Fatp5*. Data were expressed as mean ± SD (n = 10). **p < 0.01, *vs*. NG; ^#^p < 0.05, ^##^p < 0.01, *vs*. MG.

**Figure 8:**
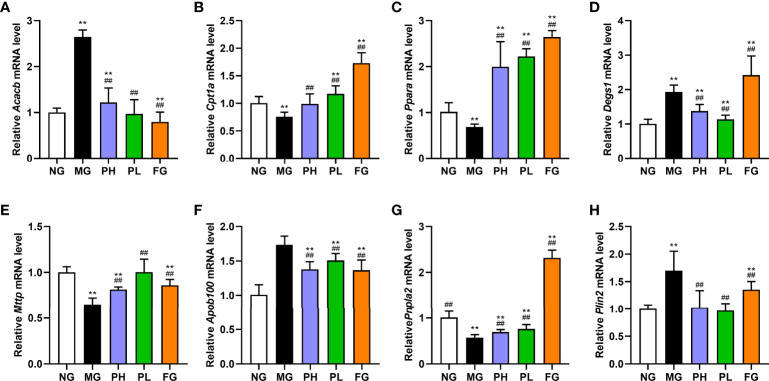
Effect of puerarin on expression of lipid metabolism-related genes in liver. **(A)** *Acacb*; **(B)** *Cpt1a*; **(C)** *Ppara*; **(D)** *Degs1*; **(E)** *Mttp*; **(F)** *Apob100*; **(G)** *Pnpla2*; **(H)** *Plin2*. Data were expressed as mean ± SD (n = 10). **p < 0.01, vs. NG; ^##^p < 0.01, vs. MG.

In view of the results of serum and liver indicators, puerarin could significantly improve liver lipid accumulation, oxidative stress, and inflammation in NAFLD rats. Therefore, the expression of key protein-coding genes in lipid accumulation pathways was examined. Compared with the NG, dietary intake of HFFD resulted in increased expressions of *Srebf1*, *Chrebp*, *Acaca*, *Fasn*, *Acacb*, *Degs1*, *Plin2*, *Scd1*, *Cd36*, *Fatp5*, and *Apob100* in rat liver (**Figure 7**, *p* < 0.01). HFFD feeding for 20 weeks also could decreased expressions of *Cpt1a*, *Ppara*, *Mttp*, and *Pnpla2* (**Figure 8**, *p* < 0.01, *vs*. NG).

In terms of *de novo* lipogenesis and fatty acid uptake, the expression levels of genes in the PH, PL, and FG were extremely reduced (**Figure 7**, *p* < 0.01, *vs*. MG). In terms of FFA β-oxidation and VLDL assembly and secretion, the expression of *Acacb*, *Apob100*, and *Plin2* in the PH, PL, and FG were markedly reversed (**Figures 8A, H**, *p* < 0.01, *vs*. MG), and the levels of *Cpt1a*, *Ppara*, *Mttp*, and *Pnpla2* genes were significantly increased (**Figures 8B, C, E–G**, *p* < 0.01, *vs*. MG). The puerarin treatment groups could evidently drop the expression level of *Degs1*; on the contrary, the expression of *Degs1* in the FG was significantly increased (**Figure 8D**, *p* < 0.01, *vs*. MG).

## Discussion

Hepatic lipid metabolic disorder is one of the main features of NAFLD, which is usually accompanied by serum lipid dyslipidemia, abnormal abdominal fat coefficients, and hepatic lipid accumulation (32). In order to better simulate the daily diet structure of modern people, HFFD is usually used to induce the establishment of a model of NAFLD. In this study, a rat model of NFALD was successfully constructed by feeding rats with HFFD for a long time, which is characterized by liver and serum lipids dyslipidemia, accompanied by abdominal obesity, hepatic oxidative stress, and inflammation. In pathological observations, a large number of fat vacuoles were produced, and inflammatory cells infiltration occurred. In addition, we found that after puerarin was added in HFFD, puerarin downregulates the fat coefficient of the abdominal cavity, suggesting that puerarin may have fat-reducing effects. Lei Yang et al. (33) found that puerarin can protect pancreatic beta cells through the GLP-1R signaling pathway, thereby reducing body weight and abdominal fat coefficient in obese model mice. After the combination of fenofibrate and HFFD, although the body weight and the abdominal fat index of the rats were improved, the long-term administration of fenofibrate may suppress the appetite of the rats, thereby reducing their appetite. On the contrary, the positive improvement effect of puerarin on final weight, index of liver and perirenal, and fat of abdominal and epididymal was not achieved by suppressing their appetite.

Serum lipid is an important indicator to measure the lipid metabolism disorder of the body. Long-term HFFD ingestion can cause an increase in serum lipid content. This *in vivo* experiment examined the effects of dietary interventions in different periods on serum lipid markers. According to the results of routine serum monitoring every 4 weeks, puerarin plays a role in improving serum lipids in week 16. After the administration of fenofibrate, the time of action seems to be shorter, and the effect is more prominent. This suggests that puerarin and fenofibrate could improve dyslipidemia in NAFLD rats. In addition, puerarin diet could lead to the secretion of more hepatic VLDL into the blood. Although puerarin excretes a large amount of hepatic VLDL, it does not cause the increase in serum TG and FFA. The mechanism of this needs to be further explored. After the experiment of rat, both high and low doses of puerarin could significantly improve the abnormality of serum FFA, TG, and VLDL.

As an important regulator of lipid homeostasis, the liver plays a central role in lipid synthesis and metabolism (34). Liver lipid accumulation is mainly determined by four mechanisms: *de novo* lipogenesis, fatty acid uptake, β-oxidation of fatty acid, and VLDL assembly and secretion (35). The contents of TC, TG, FFA, VLDL, and acetyl-CoA in the liver could clearly reflect the situation of hepatic lipid accumulation. According to our results, the pure HFFD rats had obvious lipid accumulation, which was mainly manifested as increased hepatic TC, TG, and FFA content, decreased acetyl-CoA content, blocked VLDL assembly and secretion, and liver fatty vacuoles accumulation. Therefore, this experiment further explored the mechanism of puerarin-regulating hepatic lipid from the level of gene expression. *De novo* lipogenesis and fatty acid uptake determine the rate at which lipids are synthesized by the liver. Among them, FASN (coded by *Fasn*) and ACC1 (coded by *Acaca*) are rate-limiting enzymes for *de novo* lipogenesis, SCD1 (coded by *Scd1*) is the key protein for the generation of monounsaturated fatty acids, and SREBP-1c (coded by *Srebf1*) and carbohydrate response element binding protein (CHREBP, coded by *Chrebp*) are upstream proteins regulating FASN, ACC1, and SCD1. When there is excessive fructose and fat intake, SREBP-1c and CHREBP are activated, which in turn increases the expression of genes encoding these proteins (36). Fatty acid transport protein (FATP5, coded by *Fatp5*) and cluster of differentiation 36 (CD36, coded by *CD36*) together control the rate of fatty acid uptake by the liver, and a high-fat diet significantly increases the expression of genes encoding FATP5 and CD36. In our study, puerarin significantly reduced the expression of these genes and reduced inflammation caused by lipid accumulation. β-Oxidation of fatty acid and VLDL assembly and secretion simultaneously control hepatic lipid metabolism. Adipose triglyceride lipase (ATGL, coded by *Pnplin2*) is the first rate-limiting enzyme in the degradation of TG. Long-chain fatty acids generated after TG degradation and *de novo* synthesis and acquisition of long-chain fatty acids can enter mitochondria through carnitine palmitoyl transferase 1a (CPT1A, coded by *Cpt1a*) for fatty acid β-oxidation (37). Dihydroceramide desaturase 1 (DES1, coded by *Degs1*) is the rate-limiting enzyme for ceramide synthesis, which is regulated by SREBP-1c and affects liver mitochondrial function inhibited (38, 39). Microsomal triglyceride transfer protein (MTTP, coded by *Mttp*) adds TG from the liver to apolipoprotein B 100 (APOB100, coded by *Apob100*) to form VLDL precursors (40). Finally, APOB100 secretes mature VLDL into the blood and supplies it to other organs and tissues (41). Among them, perilipin 2 family (ADRP, coded by *Plin2*) can inhibit the maturation rate of VLDL, resulting in the TG not being assembled into VLDL, which in turn accumulates in the liver. In our study, HFFD led to the reduction in the hepatic fatty acid oxidation and VLDL assembly capacity. Furthermore, the above changes could be reversed by puerarin treatment. According to the study of Guodong Zheng et al. (42), 0.2% and 0.4% puerarin mixed with HFFD had different improve effects in serum and hepatic lipids. The contents of TG and FFA in the serum and liver in 0.2% puerarin group were better than that in 0.4% puerarin group. This phenomenon is similar to our findings. Overall, in our study, 0.1% puerarin can effectively reduce HFFD-induced hepatic lipid accumulation better by modulating *de novo* lipogenesis, fatty acid uptake, fatty acid β-oxidation, and VLDL secretion.

Liver lipid overload induces overproduction of oxidants, including superoxide anion radicals (O^2−^) and hydrogen peroxide (H_2_O_2_) (43). The activities of antioxidant enzymes CAT and SOD also decrease with the development of oxidative stress (44). GSH-Px as an electron donor in oxidation and can effectively decompose H_2_O_2_ (45). AcAc is not only a product of incomplete FFA β-oxidation in the liver but can also promote the occurrence of oxidative stress (46, 47). MDA is a highly reactive aldehyde derivative produced by the combination of unsaturated fatty acids with O^2−^and H_2_O_2_, which can amplify liver damage caused by oxidative stress (43, 48). Therefore, the above-mentioned antioxidants and intermediate products of lipid oxidation can be used as important indicators to evaluate the degree of oxidative stress. When mitochondria produce a large amount of ROS due to excessive oxidation, the nuclear factor kappa-B signaling pathway will be activated, a large amount of lipotoxic substances will be produced, and then inflammatory factors will be activated (49). Lipotoxicity activates numerous inflammatory factors, such as interleukins (IL-1β and IL-18) and TNF-α (50, 51). The occurrence of inflammatory response promotes oxidative stress, thus forming a vicious circle. Finally, the two together induced liver damage. Increased AST, ALT, and ALP activities in the serum indicated that hepatocytes and their functions were damaged, i.e., hepatocyte necrosis and mitochondrial dysfunction (52). In this experiment, peroxidase, antioxidative enzymes, and hepatic function indexes were detected in the serum and liver. Compared with normal diet, HFFD developed severe oxidative stress, inflammation, and liver injury. After fenofibrate was fed to rats, the levels of SOD, CAT, GSH-Px, and MDA in the serum was decreased. However, combined with the results of hepatic inflammatory factors and liver function, fenofibrate administration seriously increased the burden of mitochondrial oxidation, resulting in a large release of inflammatory factors, which in turn caused liver injury. It was also confirmed by pathological observation that a large number of inflammatory factors infiltrated hepatocytes after taking fenofibrate. Puerarin could ameliorate hepatic oxidative stress, inflammation, and liver injury induced by HFFD. Combined with pathological observation, it was confirmed that the number of hepatic fat vacuoles and the degree of infiltration of inflammatory factors were alleviated after administration of puerarin. Low dose of puerarin has better anti-inflammatory and antioxidant effects, probably because it regulates lipid-accumulation-related pathways more significantly, so liver lipid accumulation is greatly alleviated. From this, we speculate that it may be a 0.2% dose of puerarin that exceeds its bioavailability, resulting in a decrease in its functional effects. The specific reasons for this phenomenon need to be further studied.

**Figure 9:**
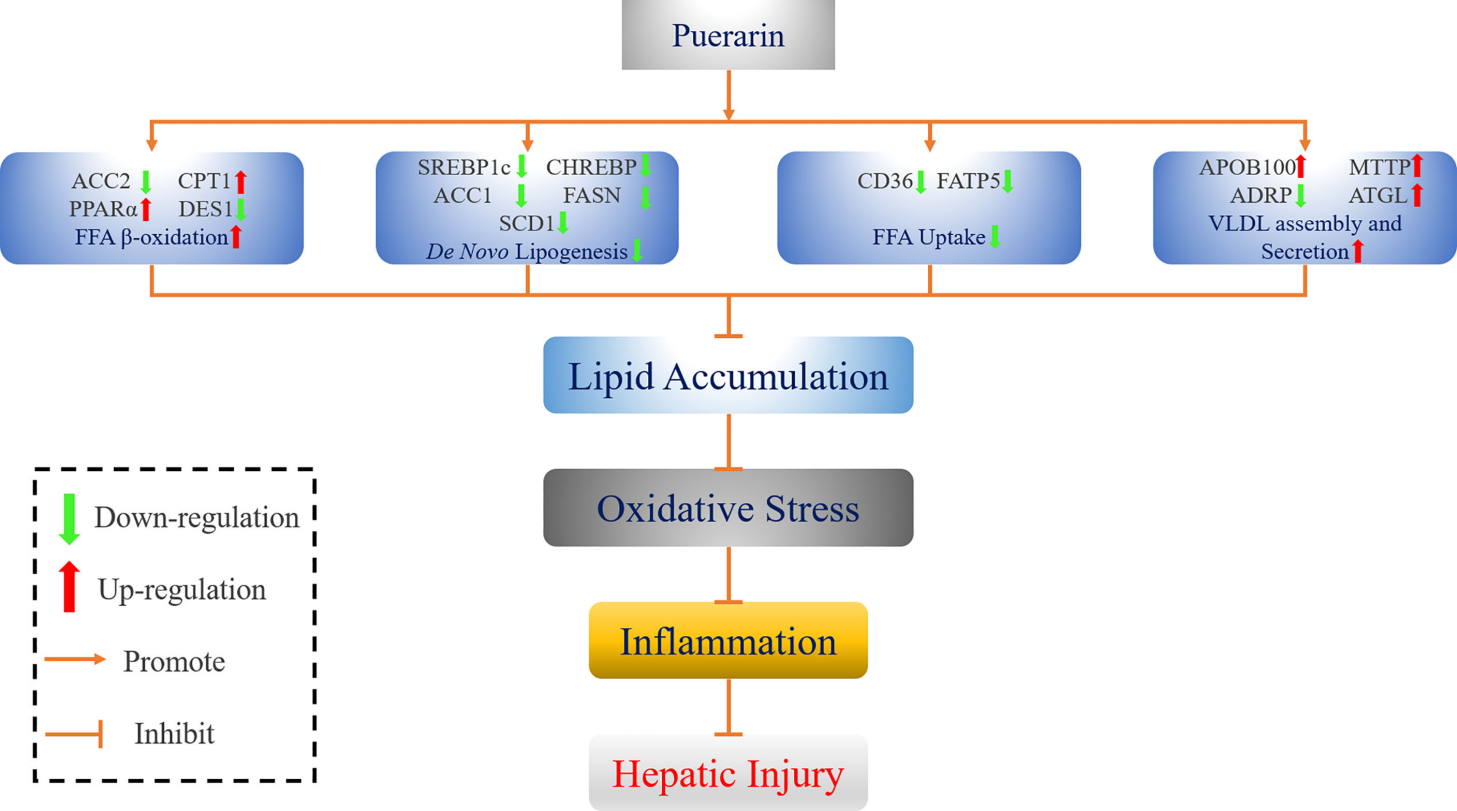
Schematic diagram of the mechanism by which puerarin ameliorates NAFLD.

In conclusion, intake of puerarin greatly ameliorated the levels of serum and liver lipid in NAFLD rats. In addition, puerarin regulated the expression of genes in *de novo* lipogenesis, fatty acid uptake, β-oxidation of fatty acid, and VLDL assembly and secretion, thereby reversing the lipid accumulation in the liver and finally improving the hepatic lipid metabolism disorder. Meanwhile, puerarin could also reduce HFFD-induced oxidative stress, inflammation, and liver injury by increasing the activity of antioxidant markers and reducing the release of inflammatory factors. **Figure 9** shows an overview of the mechanism by which puerarin ameliorates NAFLD in rats. Overall, low-dose puerarin had better positive effects. This study provides a comprehensive theoretical basis and mechanism exploration for puerarin to ameliorate NAFLD and confirms that puerarin has a certain development potential.

## Data availability statement

The original contributions presented in the study are included in the article/supplementary material. Further inquiries can be directed to the corresponding author.

## Ethics statement

This study was reviewed and approved by Animal Ethics Committee of the Beijing Key Laboratory of Functional Food from Plant Resources.

## Author contributions

JZ: investigator, writing—original draft, formal analysis, and elaborated the figures. NZ: project administration. LZ: supervision. AA and MS: funding acquisition. FZ: conceptualization, supervision, funding acquisition, and writing—review and editing. All authors contributed to the article and approved the submitted version.

## Funding

This work was financially supported by Analysis of Active Components and Function Evaluation of Danxi Hongqu Rice Wines (202205410610167) and the Taif University Researchers Supporting Project (TURSP-2020-197), Taif University, Taif, Saudi Arabia.

## Acknowledgments

We appreciate and thank Taif University for the financial support for Taif University Researchers Supporting Project (TURSP-2020-197), Taif University, Taif, Saudi Arabia.

## Conflict of interest

The authors declare that the research was conducted in the absence of any commercial or financial relationships that could be construed as a potential conflict of interest.

## Publisher’s note

All claims expressed in this article are solely those of the authors and do not necessarily represent those of their affiliated organizations, or those of the publisher, the editors and the reviewers. Any product that may be evaluated in this article, or claim that may be made by its manufacturer, is not guaranteed or endorsed by the publisher.

## References

[1] Lonardo A Byrne CD Caldwell SH Cortez-Pinto H Targher G. Global epidemiology of nonalcoholic fatty liver disease: meta-analytic assessment of prevalence, incidence, and outcomes. Hepatology (2016) 64:1388–89. doi: 10.1002/hep.28584 27038241

[2] Younossi ZM. Non-alcoholic fatty liver disease - a global public health perspective. J Hepatol (2019) 70:531–44. doi: 10.1016/j.jhep.2018.10.033 30414863

[3] Loomba R Adams LA. The 20% rule of NASH progression: The natural history of advanced fibrosis and cirrhosis caused by NASH. Hepatology (2019) 70:1885–88. doi: 10.1002/hep.30946 PMC7504908 31520407

[4] Chalasani N Younossi Z Lavine JE Diehl AM Brunt EM Cusi K. The diagnosis and management of non-alcoholic fatty liver disease: practice guideline by the American association for the study of liver diseases, American college of gastroenterology, and the American gastroenterological association. Hepatology (2012) 55:2005–23. doi: 10.1038/ajg.2012.217 22488764

[5] Medina-Santillan R Lopez-Velazquez JA Chavez-Tapia N Torres-Villalobos G Uribe M Mendez-Sanchez N. Hepatic manifestations of metabolic syndrome. Diabetes Metab Res Rev (2013) 7, 1-17. doi: 10.1002/dmrr.2410 23471889

[6] Marchesini G Bugianesi R Forlani G Cerrelli F Lenzi M Manini R. Nonalcoholic fatty liver, steatohepatitis, and the metabolic syndrome. Hepatology (2003) 38:917–23. doi: 10.1053/jhep.2003.50161 12668987

[7] Mashek DG. Hepatic lipid droplets: A balancing act between energy storage and metabolic dysfunction in NAFLD. Mol Metab (2020) 50:101115. doi: 10.1016/j.molmet.2020.101115 33186758 PMC8324678

[8] Ress C Kaser S. Mechanisms of intrahepatic triglyceride accumulation. World J Gastroenterol (2016) 22:1664–73. doi: 10.3748/wjg.v22.i4.1664 PMC4721997 26819531

[9] Kawano Y Cohen DE. Mechanisms of hepatic triglyceride accumulation in non-alcoholic fatty liver disease. J Gastroenterol (2013) 48:434–41. doi: 10.1007/s00535-013-0758-5 PMC3633701 23397118

[10] Donnelly KL Smith CI Schwarzenberg SJ Jessurun J Boldt MD Parks EJ. Sources of fatty acids stored in liver and secreted *via* lipoproteins in patients with nonalcoholic fatty liver disease. J Clin Invest (2005) 115:1343–51. doi: 10.1172/jci200523621 PMC1087172 15864352

[11] Eaton S Bartlett K Pourfarzam M. Mammalian mitochondrial beta-oxidation. Biochem J (1996) 320:345–57. doi: 10.1042/bj3200345 PMC1217938 8973539

[12] Win S Tin Aung T Le BHA Garcia-Ruiz C Fernandez-Checa JC Kaplowitz N. Sab (Sh3bp5) dependence of JNK mediated inhibition of mitochondrial respiration in palmitic acid induced hepatocyte lipotoxicity. J Hepatol (2015) 62:1367–74. doi: 10.1016/j.jhep.2015.01.032 PMC4439305 25666017

[13] Hammond LE Albright CD He L Rusyn I Watkins SM Doughman SD. Increased oxidative stress is associated with balanced increases in hepatocyte apoptosis and proliferation in glycerol-3-phosphate acyltransferase-1 deficient mice. Exp Mol Pathol (2007) 82:210–19. doi: 10.1016/j.yexmp.2006.12.004 PMC1865130 17258706

[14] Braunersreuther V Viviani GL Mach F Montecucco F. Role of cytokines and chemokines in non-alcoholic fatty liver disease. World J Gastroenterol (2012) 18:727–35. doi: 10.3748/wjg.v18.i8.727 PMC3286135 22371632

[15] Lukas Niederreiter HT. Cytokines and fatty liver diseases. Liver Res (2018) 2:14–20. doi: 10.1016/j.livres.2018.03.003

[16] Sunhyo J Miyoung H Hyunghee L Mina K Jaekwang K Christopher JN. Effects of fenofibrate on high-fat diet–induced body weight gain and adiposity in female C57BL/6J mice. Metabolism (2004) 53:1284–89. doi: 10.1016/j.metabol.2004.05.003 15375783

[17] Athyros VG Katsiki N Mikhailidis DP. NAFLD and statins. Dig Dis Sci (2020) 65:3052–53. doi: 10.1007/s10620-020-06505-x 32797344

[18] Attridge RL Frei CR Ryan L Koeller J Linn WD. Fenofibrate-associated nephrotoxicity: A review of current evidence. Am J Health Syst Pharm (2013) 70:1219–25. doi: 10.2146/ajhp120131 23820458

[19] Pinal-Fernandez I Casal-Dominguez M Mammen AL. Statins: pros and cons. Med Clin (2018) 150:398–402. doi: 10.1016/j.medcle.2018.03.001 PMC6019636 29292104

[20] Zhou YX Zhang H Peng C. Puerarin: A review of pharmacological effects. Phytother Res (2014) 28:961–75. doi: 10.1002/ptr.5083 24339367

[21] Zeng X Feng Q Zhao F Sun C Zhou T Yang J. Puerarin inhibits TRPM3/miR-204 to promote MC3T3-E1 cells proliferation, differentiation and mineralization. Phytother Res (2018) 32:996–1003. doi: 10.1002/ptr.6034 29368357

[22] Després JP Lemieux I. Abdominal obesity and metabolic syndrome. Nature (2006) 444:881–87. doi: 10.1038/nature05488 17167477

[23] Greenberg AS McDaniel ML. Identifying the links between obesity, insulin resistance and beta-cell function: potential role of adipocyte-derived cytokines in the pathogenesis of type 2 diabetes. Eur J Clin Invest (2002) 3:24–34. doi: 10.1046/j.1365-2362.32.s3.4.x 12028372

[24] Zheng P Ji G Ma Z Liu T Xin L Wu H. Therapeutic effect of puerarin on non-alcoholic rat fatty liver by improving leptin signal transduction through JAK2/STAT3 pathways. Am J Chin Med (2009) 37:69–83. doi: 10.1142/s0192415x09006692 19222113

[25] Xu WT Tang MY Wang JH Wang LH. Anti−infammatory activities of puerarin in high−fat diet−fed rats with streptozotocin−induced gestational diabetes mellitus. Mol Biol Rep (2020) 47:7537–46. doi: 10.1007/s11033-020-05816-6 PMC7588390 32946041

[26] Xu DX Guo XX Zeng Z Wang Y Qiu J. Puerarin improves hepatic glucose and lipid homeostasis *in vitro* and *in vivo* by regulating the AMPK pathway. Food Funct (2021) 12:2726–40. doi: 10.1039/d0fo02761h 33681875

[27] Zhang J Zhao L Cheng Q Ji B Yang M Sanidad KZ. Structurally different flavonoid subclasses attenuate high-fat and high-fructose diet induced metabolic syndrome in rats. J Agric Food Chem (2018) 66:12412–20. doi: 10.1021/acs.jafc.8b03574 30360615

[28] Guo XX Zeng Z Qian YZ Qiu J Wang K Wang Y. Wheat flour, enriched with gamma-oryzanol, phytosterol, and ferulic acid, alleviates lipid and glucose metabolism in high-fat-fructose-fed rats. Nutrients (2019) 11:1697. doi: 10.3390/nu11071697 PMC6683091 31340583

[29] Castiglioni L Pignieri A Fiasche M Giudici M Crestani M Mitro N. Fenofibrate attenuates cardiac and renal alterations in young salt-loaded spontaneously hypertensive stroke-prone rats through mitochondrial protection. J Hypertens (2018) 36:1129–46. doi: 10.1097/hjh.0000000000001651 29278547

[30] Zhao L Zhang N Yang D Yang M Guo X He J. Protective effects of five structurally diverse flavonoid subgroups against chronic alcohol-induced hepatic damage in a mouse model. Nutrients (2018) 10, 1754. doi: 10.3390/nu10111754 PMC6266428 30441755

[31] Zhou J Zhang N Zhao L Soliman MM Wu W Li J. Protective effects of honey-processed astragalus on liver injury and gut microbiota in mice induced by chronic alcohol intake. J Food Qual (2022) 2022:3691. doi: 10.1155/2022/5333691

[32] Rao A Kosters A Mells JE Zhang WJ Setchell KDR Amanso AM. Inhibition of ileal bile acid uptake protects against nonalcoholic fatty liver disease in high-fat diet-fed mice. Sci Transl Med (2016) 8:11. doi: 10.1126/scitranslmed.aaf4823 PMC5056562 27655848

[33] Yang L Yao DD Yang HY Wei YJ Peng YR Ding YF. Puerarin protects pancreatic β-cells in obese diabetic mice *via* activation of GLP-1R signaling. Mol Endocrinol (2016) 30:361–71. doi: 10.1210/me.2015-1213 PMC5414651 26789107

[34] Nguyen P Leray V Diez M Serisier S Le Bloc’h J Siliart B. Liver lipid metabolism. J Anim Physiol Anim Nutr (2008) 92:272–83. doi: 10.1111/j.1439-0396.2007.00752.x 18477307

[35] Ipsen DH Lykkesfeldt J Tveden-Nyborg P. Molecular mechanisms of hepatic lipid accumulation in non-alcoholic fatty liver disease. Cell Mol Life Sci (2018) 75:3313–27. doi: 10.1007/s00018-018-2860-6 PMC6105174 29936596

[36] Sanders FWB Griffin JL. *De novo* lipogenesis in the liver in health and disease: more than just a shunting yard for glucose. Biol Rev (2016) 91:452–68. doi: 10.1111/brv.12178 PMC4832395 25740151

[37] Nassir F Ibdah JA. Role of mitochondria in nonalcoholic fatty liver disease. Int J Mol Sci (2014) 15:8713–42. doi: 10.3390/ijms15058713 PMC4057755 24837835

[38] Chaurasia B Tippetts TS Monibas RM Liu JQ Li Y Wang LP. Targeting a ceramide double bond improves insulin resistance and hepatic steatosis. Science (2019) 365:386–92. doi: 10.1126/science.aav3722 PMC6787918 31273070

[39] Roszczyc-Owsiejczuk K Zabielski P. Sphingolipids as a culprit of mitochondrial dysfunction in insulin resistance and type 2 diabetes. Front Endocrinol (2021) 12:635175. doi: 10.3389/fendo.2021.635175 PMC8013882 33815291

[40] Fisher E Ginsberg H. Complexity in the secretory pathway: The assembly and secretion of apolipoprotein b-containing lipoproteins. J Biol Chem (2002) 277:17377–80. doi: 10.1074/jbc.R100068200 12006608

[41] Samata T Shadab AS. Intracellular trafficking and secretion of VLDL. Arterioscler Thromb Vasc Biol (2012) 32:7079–86. doi: 10.1161/ATVBAHA.111.241471 PMC3334296 22517366

[42] Zheng GD Lin LZ Zhong SS Zhang QF Li DM. Effects of puerarin on lipid accumulation and metabolism in high-fat diet-fed mice. PloS One (2015) 10:e0122925. doi: 10.1371/journal.pone.0122925 25822741 PMC4378957

[43] Chen Z Tian RF She ZG Cai JJ Li HL. Role of oxidative stress in the pathogenesis of nonalcoholic fatty liver disease. Free Radic Biol Med (2020) 152:116–41. doi: 10.1016/j.freeradbiomed.2020.02.025 32156524

[44] Okuno Y Fukuhara A Hashimoto E Kobayashi H Kobayashi S Otsuki M. Oxidative stress inhibits healthy adipose expansion through suppression of SREBF1-mediated lipogenic pathway. Diabetes (2018) 67:1113–27. doi: 10.2337/db17-1032 29618580

[45] Lixiao Zhang XW Cueto R Effi C Zhang Y Tan H Qin X. Biochemical basis and metabolic interplay of redox regulation. Redox Biol (2019) 26:101284. doi: 10.1016/j.redox.2019.101284 31400697 PMC6831867

[46] Shi XX Li DD Deng QH Peng ZC Zhao CX Li XB. Acetoacetic acid induces oxidative stress to inhibit the assembly of very low density lipoprotein in bovine hepatocytes. J Dairy Res (2016) 83:442–46. doi: 10.1017/s0022029916000546 27692001

[47] Kadir AA Clarke K Evans RD. Cardiac ketone body metabolism. Biochim Biophys Acta Mol Basis Dis (2020) 1866:165739. doi: 10.1016/j.bbadis.2020.165739 32084511

[48] Serviddio G Bellanti F Vendemiale G. Free radical biology for medicine: learning from nonalcoholic fatty liver disease. Free Radic Biol Med (2013) 65:952–68. doi: 10.1016/j.freeradbiomed.2013.08.174 23994574

[49] Park J Min JS Kim B Chae UB Yun JW Choi MS. Mitochondrial ROS govern the LPS-induced pro-inflammatory response in microglia cells by regulating MAPK and NF-κB pathways. Neurosci Lett (2015) 584:191–6. doi: 10.1016/j.neulet.2014.10.016 25459294

[50] Mulati A Zhang X Zhao T Ren B Wang LF Liu XN. Isorhamnetin attenuates high-fat and high-fructose diet induced cognitive impairments and neuroinflammation by mediating MAPK and NF kappa b signaling pathways. Food Funct (2021) 12:9261–72. doi: 10.1039/d0fo03165h 34606526

[51] Liu Q Ma RS Li S Fei YJ Lei J Li RY. Dietary supplementation of auricularia auricula-judae polysaccharides alleviate nutritional obesity in mice *via* regulating inflammatory response and lipid metabolism. Foods (2022) 11:18. doi: 10.3390/foods11070942 PMC8997552 35407029

[52] Zhao L Wang Y Liu J Wang K Guo XX Ji BP. Protective effects of genistein and puerarin against chronic alcohol-induced liver injury in mice *via* antioxidant, anti-inflammatory, and anti-apoptotic mechanisms. J Agric Food Chem (2016) 64:7291–97. doi: 10.1021/acs.jafc.6b02907 27609057

